# Chest wall perforator flap partial breast reconstruction: a retrospective analysis of surgical, cosmetic and survival outcome

**DOI:** 10.3332/ecancer.2024.1681

**Published:** 2024-03-14

**Authors:** Sanjit Kumar Agrawal, Shagun Mahajan, Rosina Ahmed, Neela Shruti, Abhishek Sharma

**Affiliations:** Department of Breast Oncosurgery, Tata Medical Center, Kolkata 700156, India; * Equally contributed

**Keywords:** breast cancer, breast conservation surgery, chest wall perforator flap

## Abstract

**Introduction:**

Oncoplastic breast surgery includes volume replacement as well as volume displacement. Autologous tissue is the preferred approach for volume replacement and includes chest wall perforator flaps (CWPF). Although described more than a decade ago, CWPFs have not been adopted widely in clinical practice till recently. We report the largest single-centre institutional data on CWPFs.

**Patients and methods:**

The study includes all patients who underwent breast conservation surgery (BCS) using CWPFs from January 2015 to December 2022. Data were retrieved from the institutional electronic record and Redcap database. The analysis was done using SPSS 23 and STATA 14.

**Results:**

150 patients were included in the study. The mean age was 48.8 years (SD 10.4), and the body mass index was (26.6 kg/m^2^, SD 4.3). >50% of patients had breasts with small cup sizes (A&B) and mild ptosis (Non-ptotic and Grade 1 ptosis). 44.7% of patients underwent lateral intercostal artery perforator flap (LICAP), anterior intercostal artery perforator flap in 31.3%, lateral thoracic perforator flap (LTAP) in 12%, LICAP + LTAP in 11.3% and thoracodorsal artery perforator flap in 1%. Post-operatively, haematoma was seen in 1.3%, complete flap necrosis in 1.3%, seroma in 7%, wound dehiscence in 12%, and positive margin in 6.7%. 92 patients responded to the satisfaction assessment, of which >90% were happy with the surgical scars, comfortable going out in a public place, satisfied with the symmetry of the breast, and no one chose mastectomy in hindsight. The 5-year predicted disease free survival and overall survival were 86.4% and 94.7%, respectively.

**Conclusion:**

BCS with CWPF is an excellent option for reconstruction in small to medium-sized breasts. It is associated with minimal morbidity and comparable patient-reported cosmetic and survival outcomes.

## Introduction

Breast conservation surgery (BCS) is routinely performed in early breast cancer patients who desire to retain their breasts. There is extensive evidence that it is oncologically safe, and survival outcomes are similar to mastectomy [1]. Some recent population-based studies suggest that long-term survival of BCS with radiation may even be superior to mastectomy [2, 3].

Oncoplastic breast surgery includes volume displacement and volume replacement techniques [4]. Volume replacement is required to fill the breast defect of patients with a small to moderate breast size in whom wider resection is required because of a large tumour size. The latissmus dorsi (LD) flap was traditionally used for partial breast reconstruction. However, removing a major muscle increases morbidity with shoulder stiffness and donor-site seroma and has the potential to impair functional outcomes [5, 6]. Chest wall perforator flaps (CWPF) are muscle-sparing fascio-cutaneous flaps which have become increasingly popular as a choice of surgery in such patients. The advantages include avoiding a mastectomy and providing skin cover for reconstruction if needed. The CWPF procedure is less morbid, and complications like seroma, wound dehiscence and loss of shoulder function are less compared to LD flap [7].

Angrigiani *et al* [8], first reported using a cutaneous island of LD flap based on a single cutaneous perforator for reconstruction, sparing the LD muscle. Hamdi *et al* [9] first described using a lateral intercostal artery perforator (LICAP) flap in 2004, but use was restricted to filling lateral breast defects because of the short pedicle. McCulley *et al* [10] described that a lateral thoracic artery perforator (LTAP) flap can be used exclusively or in combination with a LICAP for partial breast reconstruction. LTAP allows greater mobilisation and a larger flap size than LICAP. The anterior intercostal artery (AICAP) flap has also been described to cover the lower quadrant defects [11].

To our knowledge, this study is the largest single institutional study on CWPF and is an extension of our previous small case series published in 2020 [12]. We report various parameters of the CWPF reconstruction procedure, such as surgical details, complications, patient satisfaction and survival outcomes.

## Patients and methods

The breast cancer patients who underwent BCS along with immediate CWPF reconstruction from January 2015 to December 2022 at our institute were included in this study. Data on clinicopathological characteristics, surgical details and complications, adjuvant treatment and follow-up were retrieved from hospital management system software and prospectively maintained REDCap database. The institutional ethics committee approved the study via reference EC/WV/TMC/014/19.

Pre-operative markings of breast band size and cup size were noted in a standing position. Perforators (from the AICAP/posterior intercostal artery and/or thoracodorsal artery) were marked based on surgical planning and anticipated breast defect. A handheld Doppler was used to identify the perforators. These were confirmed after the patient was laid in a supine (sandbag behind the ipsilateral shoulder) position. In most cases, single-incision and supine positions were used for wide local excision and reconstruction. A specimen mammogram or intraoperative ultrasonography (in upfront surgery patients with obvious clinically palpable tumours) was done after BCS to confirm negative margins. Axillary procedure (Sentinel lymph node biopsy and/or axillary dissection) was performed through the same incision except for patients undergoing AICAPs. Flaps were de-epithelized before filling the cavity if the skin was not excised during wide local excision. Flaps were either rotated or flipped carefully without twisting the perforators or used as propeller flaps with the donor site skin.

In some cases, indocyanine green (ICG) dye (1 mL) was given intravenously using a fluorescence infra-red hand camera (Irrilic PVT Ltd) to confirm the vascularity of the flap. Drain was used only in patients who underwent axillary dissection. Patients were discharged the next day and reviewed in the breast surgery clinic within a week and 3 weeks post-surgery. All surgical complications occurring up to 90 days after surgery were recorded and analysed. The post-surgical histopathology reports were discussed in the multi-tumour board to discuss the adjuvant treatment plan.

The data were represented using summary statistics of number, percentage, mean and standard deviation. An acquired-informal questionnaire using a 4-point Likert scale was created and used to assess patient satisfaction at least 6 months after completion of radiotherapy. The survival analysis was done using Kaplan-Meier graphs. The SPSS 25 and Stata 14 versions were used for statistical analysis.

## Results

One hundred fifty patients were included in the study. 114 (76%) of the patients had upfront surgery and 25 (17%) were diabetic. >50% of patients had small cup size (A&B) and mild ptosis (non ptotic and grade 1 ptosis). The post-wide local excision defects were mainly in the lateral quadrant (72%). The demographic and histopathological details are summarised in Table 1.

We summarised surgical details in Table 2. LICAP (44.7%) was the most common flap, followed by AICAP (31.3%). Ten patients have positive margins, nine had cavity excision, and one had undergone a completion mastectomy due to persistent positive margins.

Two patients had a total flap loss, and five had marginal flap necrosis, managed by debridement and secondary suturing. 13 (6.7%) had seroma, mainly in patients having axillary lymph node dissection (ALND) and only 2 (1.3%) had post op hematoma. 19 (12.7%) had minor wound dehiscence; all were managed conservatively. The surgical site infection and post-op antibiotic use rates were 8.7% and 10.7%, respectively.

On the patient-reported outcome questionnaire survey (Table 3), >90% were happy with the surgical scars, comfortable going out in a public place, and satisfied with the symmetry of the breast. No one, in retrospect, reported having a feeling that they should have opted for a mastectomy compared to BCS.

**Figure 1.**
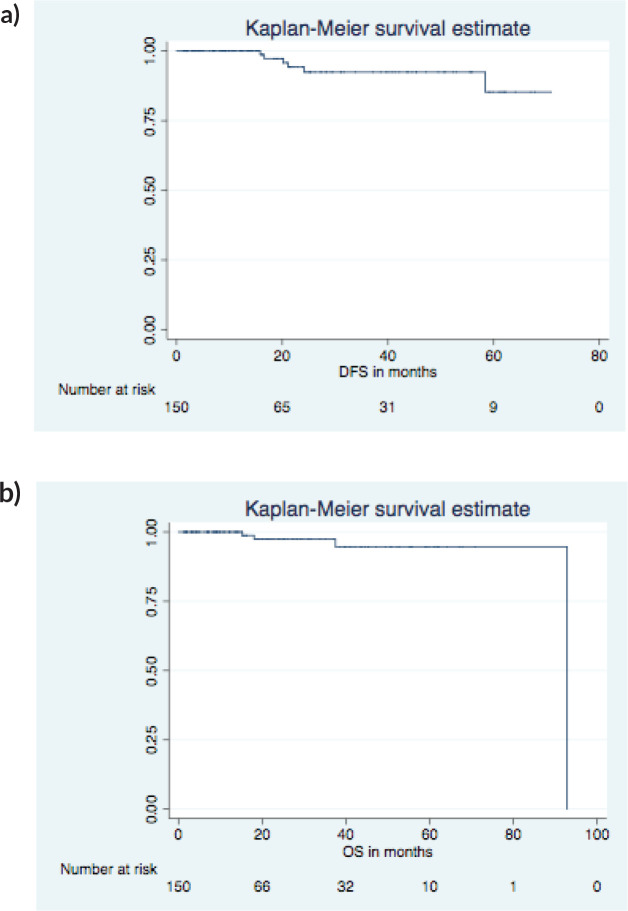
5 years predicted DFS and OS. (a): DFS KM graph. (b): OS KM graph.

**Figure 2.**
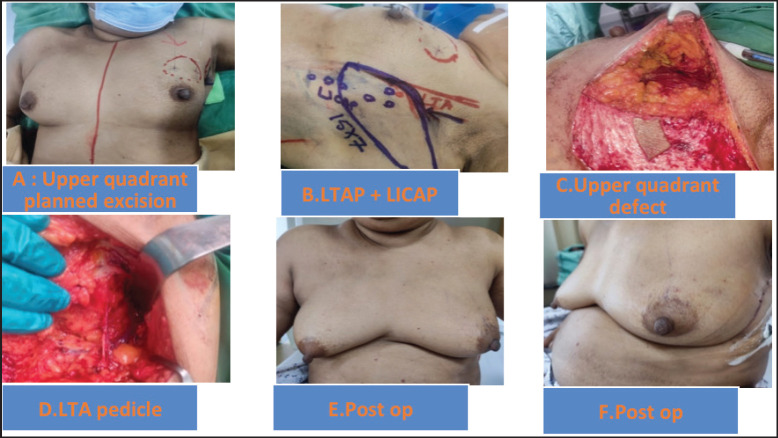
(a–f): Upper quadrant defect LTAP+ LICAP flap.

**Figure 3.**
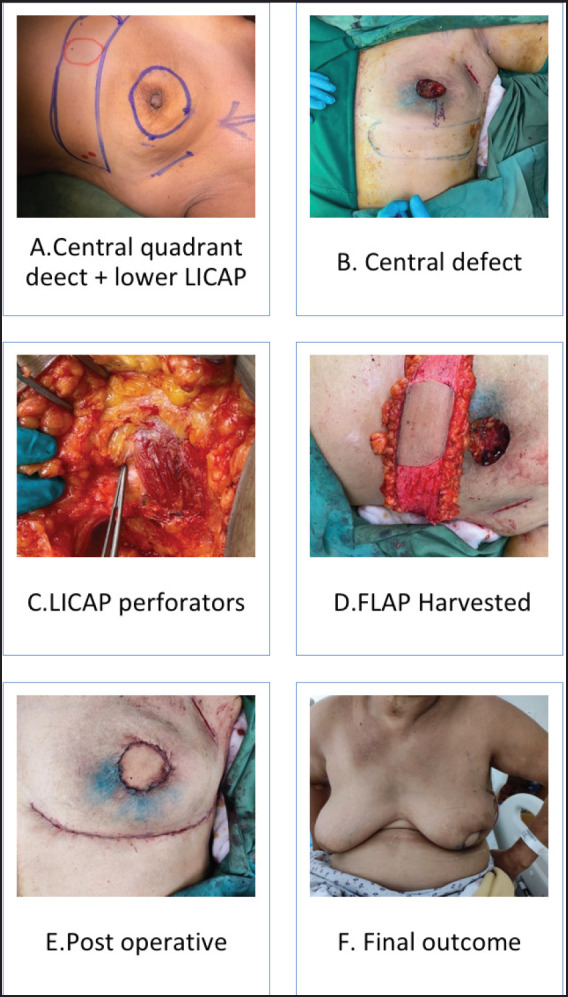
(a–e): Lower LICAP (Based on 6th and 7th intercostal perforators for large central quadrant defect).

On a median follow-up of 16 (IQR 9-37) months, 6 had recurrence (4 distant, 2 local and distant) and 4 died (3 disease progression, 1 chemotoxicity). The 5 years predicted disease free survival (DFS) and overall survival (OS) (Figure 1a and b) were 86.4% and 94.7%, respectively. The surgical images of LICAP + LTAP and lower LICAP are represented in Figures 2 and 3.

## Discussion

To our knowledge, our study is the largest single-centre institutional study on CWPF-based patrial breast reconstruction. The CWPF surgical procedure in our cohort could be used to cover the defects of all quadrants of breasts. We report an acceptable complication rate, high patient satisfaction and comparable survival rates.

**Figure 4.**
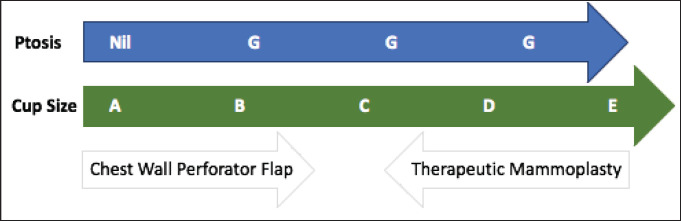
CWPF: A&B cup-size breasts with no or mild ptosis. Therapeutic mammoplasty (TM): D cup and above size with grade 2 or 3 ptosis. CWPF or TM: For C-cup-size breasts depending upon the breast ptosis, excised volume and location of the defect.

The patient selection is crucial for the successful CWPF partial breast reconstruction. It depends upon breast size, ptosis grade, location and extent of the excised breast volume [13]. In our cohort, The CWPF procedure was mainly offered for patients with small to moderate size breasts with no or mild ptosis. In the larger breast size with ptosis, therapeutic mammoplasty is a better option. We followed a simple algorithm, as shown in Figure 4. For large-volume defects, CWPF is a better option in A&B cup-size breasts with no or mild ptosis, whereas therapeutic mammoplasty can be a better option for D cup and above sizes with grade 2 or 3 ptosis. For C-cup-size breasts, both CWPF and TM could be an option depending upon the breast ptosis, excised volume and location of the defect. When initially described, CWPF were used mainly for the lateral quadrant defects [11]. however, we have used the CWPFs for the defects in all quadrants, with good cosmetic outcomes. LICAP was used to cover lateral and/or central quadrant defects (Figure 3), LTAP (Figure 2) with greater flap mobility was used for lateral and/or upper inner quadrant defects, and AICAP was used for lower quadrant defects.

The flap harvest incision planning depends upon the location and extent of the wide local excision defect. In our early case series published in 2020, we used a transverse fusiform flap design, as described by Hamdi and McCulley *et al* [9–11]. Later, to increase the volume of flap harvest, we adapted the lazy s/longitudinal flaps with the advantage of easy access to all breast quadrants with hidden scars in the axilla and inframammary line. A similar technique was published by Meybodi *et al* [14]. Some authors have advocated a two-stage procedure (initially wide local excision, followed by definitive CWPF reconstruction after confirmation of margin negativity on HPE report) in anticipation of the difficulty of margin revision with flap *in situ* [15]. We did one stage (wide local excision + CWPF) in all patients with a margin positivity rate of 6.6%. In our experience, the re-exploration for the margin revision was relatively easy, as pillar mobilisation is generally not done in patients planned for volume replacement mammoplasty.

The multicentric PartBreCon Study reported surgical complications such as hematoma and wound infection in 4.3% of patients and flap loss in 0.6% of patients [16]. Our cohort’s hematoma rate was low (1.3%), and wound infection rate was 8.7%. We noticed minor wound dehiscence in 12.7% of patients, possibly due to long scars associated with CWPF. The seroma rate was 8.7%, mainly in patients with ALND. All these complications were managed conservatively. Two patients (1.3%) in the first fifty had complete flap loss. Following this, the interoperative Doppler was used to confirm the perforators, and fluorescence mapping of the flap was used to reduce the flap necrosis complication.

The Oxford University Hospital, prospective cohort study, reported patient satisfaction using Breast – Q score with a physical well-being score of 75%, psychological well-being of >80%, sexual well-being of 60% and a physical discomfort score of 80% [17]. Additionally, at a median follow of 4.5 years, the DFS was 86%, and the OS was 94.8%. Our study shows the same trend of high satisfaction scores with the procedure and very similar 5-year predicted DFS and OS of 86.4% and 94.7%, respectively.

Our study has some inherent limitations. Being retrospective, the analysis may be associated with the selection bias. The follow-up of 18 months is short to draw any conclusion on survival outcomes. Additionally, we have used an acquired Likert’s scale questionnaire for patient-reported outcomes, which is not validated and a standard tool like the Breast Q score.

## Conclusion

The CWPF is an additional valid oncoplastic breast surgery option for mild to moderate size non-ptotic breasts with large volume wide local excision. In our experience, one-stage CWPF can cover all quadrant WLE defects with acceptable margin positivity and surgical complication rate. The patient satisfaction and survival outcomes look promising in early follow-up.

## Conflicts of interest

The authors declare no conflict of interest for this study.

## Funding

None.

## Figures and Tables

**Table 1.** Demographics and histopathological details.

Parameter	Mean (SD), *N* (%)
Age (*n* = 150)	48.8 (10.4) years
BMI (*n* = 150)	26.6 (4.3) kg/m^2^
Diabetes (*n* = 150) Yes No	25 (17%) 125 (83%)
First treatment (*n* = 150) Upfront surgery NACT	114 (76%) 36 (24%)
Cup size (*n* = 150) A B C D E	10 (6.7) % 74 (49.2%) 52 (34.7%) 13 (8.7%) 1 (0.7%)
Ptosis (*n* = 150) Non ptotic Grade 1 Grade 2 Grade 3	55 (36.7%) 63 (42%) 26 (17.3%) 6 (4%)
Tumour quadrant (*n* = 150) UOQ UIQ LOQ LIQ Central Multicentric	77 (51.3%) 4 (2.7%) 31 (20.7%) 28 (18.7%) 8 (5.3%) 2 (1.3%)
Diagnosis (*n* = 150) DCIS IDC ILC Other cancer Phyllodes Adenomyoepithelioma	4 (2.7%) 132 (88%) 3 (2%) 8 (5.3%) 2 (1.3%) 1 (0.7%)
ER (*n* = 143) Positive Negative	117 (81.8%) 26 (18.2%)
PR (*n* = 143) Positive Negative	105 (73.4%) 38 (26.6%)
Her2 receptor (*n* = 142) Positive Negative Equivocal	20 (14.1%) 83 (58.5%) 39 (27.%%)

NACT, Neo adjuvant chemotherapy; UOQ, Upper outer quadrant; UIQ, Upper inner quadrant; LOQ, Lower outer quadrant; LIQ, Lower inner quadrant; DCIS, Ductal carcinoma *in situ*, IDC, Invasive ductal cancer; ILC, Invasive lobular cancer; ER, Estrogen receptor; PR, Progesterone receptor

**Table 2.** Surgical and adjuvant treatment details (*n* = 150).

Parameter	Mean (SD), *N*(%)
Type of flap LICAP LTAP LICAP+LTAP AICAP TDAP	67 (44.7%) 18 (12%) 17 (11.3%) 47 (31.3%) 1 (0.7%)
Axilla surgery SLNB ALND	65 (43.9%) 83 (56.1%)
Flap Dimension (L)	12.4 (3.2) cm
Flap Dimension (W)	6.9 (2) cm
Specimen weight (g)	130 (46) gm
Surgery time (minute)	164 (37) mins
pT (cm)	2.9 (1.3) cm
Post-operative radiological margin assessment Specimen Mammography Intraoperative ultrasound	59 (39.3%) 91 (60.7%)
Margin Negative Positive	140 (93.3%) 10 (6.7%)

LICAP, Lateral intercostal artery perforator; LTAP, Lateral thoracic artery perforator; AICAP, Anterior inteCostal artery perforator; TDAP, Thoracodorsal artery perforator; ALND, Axillary lymph node dissection; SLNB, Sentinel lymph node biopsy

**Table 3.** Patient-reported outcome (*n* = 92).

	Likert’s scale answer: 1: Highly dis-satisfied, 2: dis-satisfied, 3: satisfied, 4: highly satisfied			
Questions	1	2	3	4
How satisfied are you with your scar?	0	1 (1.1%)	3 (3.2%)	88 (95.7%)
How comfortable are you going out in public?	0	1 (1.1%)	4 (4.3%)	87 (94.6%)
How happy are you with your treated breast in comparison to opposite breast?	0	4 (4.3%)	19 (21.7%)	69 (75%)
Do you feel in retrospect, that you should have opted for mastectomy?	0/92			
